# Coaching adaptive skill and expertise in Premier League football academies—paving a way forward for research and practice

**DOI:** 10.3389/fspor.2024.1386380

**Published:** 2024-04-10

**Authors:** Michael Ashford, Jamie Taylor, Danny Newcombe, Áine MacNamara, Stephen Behan, Simon Phelan, Scott McNeill

**Affiliations:** ^1^ Insight SFI Research Centre for Data Analytics, Dublin City University, Dublin, Ireland; ^2^ Moray House School of Education and Sport, The University of Edinburgh, Edinburgh, United Kingdom; ^3^ School of Health and Human Performance, Faculty of Science and Health, Dublin City University, Glasnevin, Ireland; ^4^ Coach Development, The Premier League, London, United Kingdom; ^5^ Department of Sport, Health Sciences and Social Work, Oxford Brookes University, Oxford, United Kingdom

**Keywords:** Premier League, football academies, expertise, skill, coaching

## Abstract

Within the domain of coach education researchers have long called for a paradigm shift, whereby the quality of coaching practice is no longer measured against a checklist of prescribed competencies. This desire to evolve coach education and development, has been aligned to the need to better identify, understand and utilise what adaptive skill and expertise looks, sounds and feels like across specific sport coaching contexts. This paper outlines a broader research plan for the Premier League to drive the progress of research informed practice, in turn shaping a coach development agenda focused on developing adaptive and skilful coaches within Academies. In turn, this is a core feature of the Premier Leagues institutional aim of developing the most skilful coaches in the world. However, in order to begin the process of initiating such a shift in the way things work, there is a need to seek first to understand, before being understood. Therefore, to demonstrate an evidence informed basis to this shift within coach education and development, we ask three questions; (1) Do we understand what the coaches with the highest level of expertise can do? (2) How should we identify coaches with expertise across different contexts? (3) What does *coaching expertise* research need to do? In answer to these questions, we present the lack of empirical investigation previously conducted in the sports coaching discipline to explore coaching expertise and draw on wider domains to offer possible capacities of skilful coaches who possess expertise. To identify coaches with expertise, coherent with the broader expertise literature, we suggest that this is best conducted via means of social validation. Finally, we offer a road map of investigation designed to explore expertise, formed of a mix of evidence informed methodologies which have not yet been utilised in sport coaching research.

## Introduction

The financial and cultural centrality of sport, and football in particular, has seen sports coaching receive significant and ongoing attention across the world. The Premier League's institutional aim is to stage the most competitive and compelling football league in the world, showcasing the most skilful football players from across the globe. Aligned to this intention, the Premier League is committed to generating domestic potential. In their most recent EPPP 10-year report (1) it was reported that 77% of professional contracts across the Premier League and EFL are held by Home Grown Players. Additionally, since 2012/13, 47% players who have featured in the Premier League have been Home Grown and the percentage of minutes from home grown players has grown from 46.05%–50.15% in the 2020/21 season. Therefore, a core component of the Premier League *virtuous circle* business model is the strategic and responsible reinvestment of broadcast and commercial revenue to support the clubs, with the aim of continuing to produce more and better home-grown players. Furthermore, the Premier League have deployed a variety of strategies towards this overall aim such as the Elite Player Performance Plan (EPPP), first introduced for football academies in 2012. The EPPP is a long-term plan that promotes the development of a world-leading Academy system, designed to best prepare world class youth players for playing in the Premier League (2). A core strand of the EPPP is the parallel development of coaches in the academy system, with the strategic aim of developing the most skilful coaches in the world (3). To advance this agenda, it is crucial to define highly skilful coaching, to understand the development of expertise, and to explore why some are recognised as experts by their peers.

What characterises expertise in coaching has been an area of interest in the sport coaching literature since the 1990's (4–6). Early investigations into skilful coaching practice looked to record and quantify patterns of coaching behaviour, which have since been employed to explore “top level professional” football coaches in academy settings (7–10). These authors have often employed systematic observation methods to investigate the self-awareness of coaches by investigating the alignment between what coaches say they intend to do vs. their behaviours in practice (7). Whilst this self-awareness is useful for coaches, if we are to truly explore coaching skill and expertise, there is an opportunity to move beyond systematic behavioural observation that captures what coaches do (11). To achieve this, research should aim to capture, make sense of, and appreciate the day to day challenges of skilful academy football coaches, alongside the contextual skills, attributes, knowledge and experiences required to solve them. Unfortunately, this type of research has not yet been conducted within football, nor the wider coaching literature.

In contrast, the broader field of expertise literature shows a rich tradition of appreciative inquiry into the skill of practitioners with high levels of expertise and comparison with novices (12–17). The notion of appreciative inquiry employs methods which emphasise the skill and expertise of individuals, rather than focusing on what needs fixing within a domain (18). In this sense, Hoffman (19) appreciates an individual with expertise to be is someone who is;

Highly regarded by peers, whose judgments are uncommonly accurate and reliable, whose performance shows consummate skill and economy of effort, and who can deal effectively with certain types of rare or “tough” cases. Also, an expert is one who has special skills or knowledge derived from extensive experience with subdomains (p. 85).

This definition captures the necessity for individuals to frequently adapt skilfully to the unique demands of their domain (*which we will explore more later in this paper*). Parallels can be drawn to Herbert Simon's metaphor of the scissors, where human behaviour is shaped by the twin blades of task environments and the capacities of the individual actor to solve them (20). In following this metaphor, empirical research in sport coaching seems to only scratch the surface of the twin blades of skilful coaching. Therefore, in this article we aim to progress the notions of skill and expertise in sport coaching using both literature from within the research field of sport coaching and externally. Specifically, we will consider three main questions;

1. Do we understand what the coaches with the highest level of expertise can do?
2. How should we identify coaches with expertise across different contexts?
3. What does coaching expertise research need to do?

## Do we understand what the coaches with the highest level of expertise can do?

The field is rich with conceptual frameworks of expertise, particularly highlighted by the seminal works of Nash et al. (21) and Lyle & Cushion (22), which provide a foundational basis for our exploration into coaching expertise. Both papers employ a narrative review of prior literature, within and external to sport coaching. Flowing from their review of literature was a deeper parameterisation of coaching expertise (see Table 1). A pivotal theme in both papers is the conditional application of knowledge to solve daily challenges, which we will explore later as a central feature (23). Both papers draw on the challenge of identifying individuals who meet the threshold of expertise or skill based on hierarchical status, years' of experience, the quality of athlete they worked with, or the success of their athletes. Instead, Nash and colleagues (21) extend this idea to consider three indicators of expertise, *experience, knowledge, and reputation*.

**Table 1:** Parameterisation of coaching expertise according to prior reviews of literature.

Nash et al. (21)	Lyle & Cushion (22)
Utilise a large declarative knowledge base when problem solving and making decisions.	Has a well-formed philosophy which supports them to solve problems effectively.
	Situational judgment and decision making.
Utilises heightened perceptual skills, mental models, sense of typicality and routines.	Has prolonged experience of the day-to-day role they have.
	Understands the demands of the context in which they work.
Effective reflection skills and a lifelong learning attitude to their development.	Heightened intellect and understanding.
Can work independently & are capable of producing novel, innovative solutions.	Possesses personal qualities which supports them to communicate effectively.
Takes their own strengths, but also limitations into account (and is aware of where these are).	Functional skills (anything not technical/tactical and sport related).
Can manage complex planning processes in light of complex problems.	Sport specific knowledge.
	Specialised knowledge (e.g., psychology, physiology).
Has a track record of “performance” within their field.	

## What can experts do—an expertise lens

There is a clear, whilst conceptual argument which is consistent across the coaching expertise literature (24–27). Universally, authors are suggesting that coaching expertise is characterised by the capacity for adaptation to context (23). In the broader literature, this capacity for change is made in the distinction between routine expertise and adaptive expertise, first made by Hatano and Inigaki (28). At the heart of both adaptive and routine expertise is that coaches can perform standard tasks and functions without error. Routine expertise captures the coach's competence in tasks with the ability to present work with low variability in the deliverables of performance. For example, the coach with routine expertise would be capable of delivering similar sessions to similar groups of athletes based on similar scheduling of activities, progressions, interactions, and time scales. They would in essence be highly effective in predictable circumstances. Conversely, the adaptive expert can produce the routine, but is also efficient and innovative in applying knowledge when approaching everyday challenges common to their job role (28, 29). Thus the adaptive expert would not only be able to generate appropriate plans for different individuals and groups, but would also be able to deviate from what was planned based on changing circumstance, with the plan acting as a useful guide.

Evidence from other domains would suggest that the skilful capacity to utilise flexible, creative, and innovative use of the competencies found in routine expertise, is what enables adaptability in practice. For instance, within the context of outdoor education, instructors with expertise have demonstrated heightened situational awareness and the capacity to recognise the demands of their environment through cues (30). The capacity to notice key information results in loops of perception, comprehension, and projection, which supports experts to filter salient information to make optimal decisions which diverge away from original plans of action (31, 32). Thus, following the guidance of others, adaptability is the essential ingredient for expertise to be realised (33, 34).

**Figure 1:**
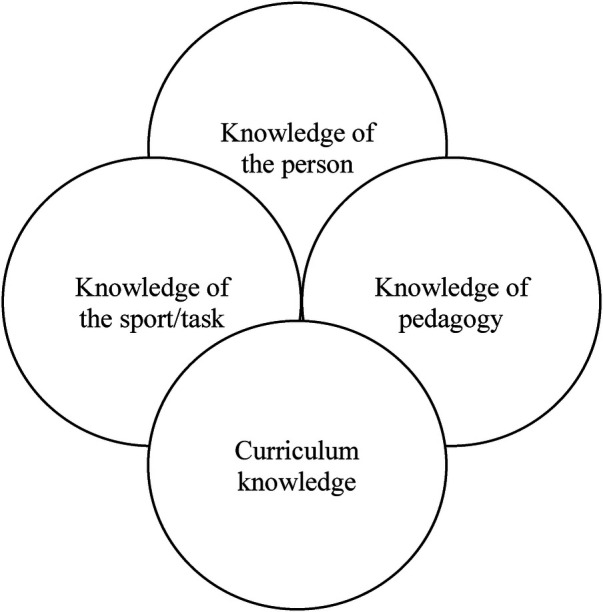
Bodies of sport coaching knowledge [adapted from (35)].

Interestingly, research has suggested that expertise is often aligned to individuals who have a wider knowledge base. In the context of coaching this refers to the amalgamation of knowledge of the person they are coaching, the sport, their pedagogy, and the curriculum they create (35, 36; see Figure 1). However, Gary Klein (37) has suggested this is a misinterpretation of the relationship those with expertise have with knowledge, which is nicely summarised by Ward et al. (38) below;

A shift away from knowing more, toward thinking dynamically, innovatively, and differently—knowing when and when not, and knowing how and why, to generate new solutions on the fly in the face of adversity and anomalies.

For example, a skilful football coach might notice that individual players have not yet developed the technical competence and skill to successfully execute a desired tactical solution within their agreed way of playing. The skilful coach may then apply knowledge of the person and pedagogy to effectively support these individuals based on the contextual and critical application of this knowledge, where other coaches may have resorted to solutions based on sharing more knowledge from a single area.

This is not to say that skilful adaptive coaches simply ‘turn up and adapt' quite the contrary! Instead, as someone engages in deliberate thinking regarding their projected decision making, the more they enhance their capacity for intuitive thinking in the moment (36). Deliberate thinking supports someone to increase the number of options available to them coupled with a greater depth of critical thought regarding which option, where, when and why, particularly when addressing problems “in the moment” through intuitive thinking. This is nicely captured by the pithy phrase “what you know determines what you see” (39). The role of knowledge therefore (e.g., the when's, when nots, how's and why's) can be captured by the metaphor of a snake shedding their skin. In essence, the use of knowledge is just as much about unlearning as learning to tackle dynamic anomalies (40, 41). This efficient combination and application of knowledge to specific environments over time characterise the expression of expertise. In sport, this requires coaches to combine a range of bodies of knowledge (see Figure 1) that are most applicable to typical and atypical challenges (35, 42, 43). Similarities can be drawn to work on conceptions of knowledge, where individuals move from dualistic, through to increasingly relativistic views based on increasing volumes of valid experience (44, 45).

Of note, more recent work has suggested that the separation of routine and adaptive expertise is unnecessary, as adaptive skill is essentially the central feature of expertise, rendering the distinction redundant (33). Instead, Ward and colleagues defined adaptive skill as;

Timely changes in understanding, plans, goals, and methods in response to either an altered situation or updated assessment of the ability to meet new demands, that permit successful efforts to achieve intent…or successful efforts to realize alternative statements of intent that are not inconsistent with the initial statement but more likely to achieve beneficial results under changed circumstances (33)

**Figure 2:**
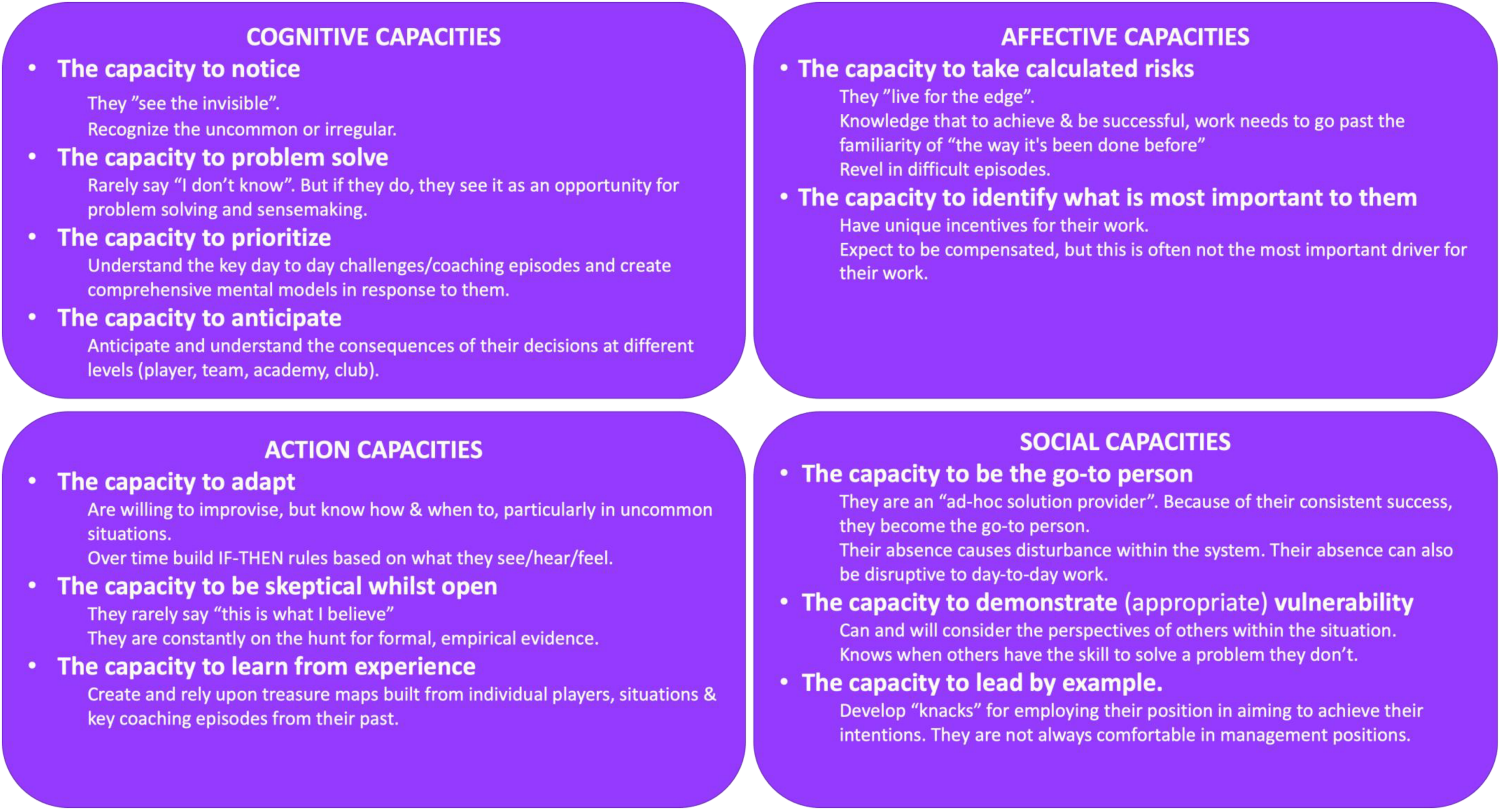
The proposed cognitive, affective, action and social capacities of academy football coaches with a high level of expertise [adapted from (46)].

This intriguing definition illustrates that we cannot separate the skill and expertise of a person from coaching episodes within their context. In this sense, evidence from the domain specific expertise literature strongly implies that skill and expertise is contextual to a person's role, responsibilities, and wider system in which they work (46). Furthermore, interpretation of this body of literature suggests that skill and expertise is characterised by several key cognitive, affective, action and social capacities which are presented, and defined in Figure 2 (46). Thus, these capacities demonstrate how complex it is to (i) identify the most skilful coaches and (ii) go about investigating and exploring these altered and updated episodes.

### The role of context

These more recent definitions of adaptive skill and expertise, alongside the capacities presented in Figure 2, draw our attention to the centrality of context dependency. A “one size fits all” expert is not to be found, especially in sport coaching with dramatic differences in role frame based on the age or stage of the participant (47, 48), their motivations (49), the values and norms of the sport, and ultimately any other conceivable contextual difference. In the context of coaching within Premier League academies, this would suggest that skilful coaching is not only different across phases of development, but also across clubs, age groups, and even with different players. The dynamic nature of Academy Football coupled with the complexities of the role presents coaches with a significant range of challenges daily. Therefore, there is a requirement to be adaptive daily within any role. Put simply, context is key (50, 51). For those interested in the development of expertise, this clearly presents a significant challenge. For the Premier League to investigate the adaptive skill and expertise of the most skilful academy coaches, there needs to be a contextual appreciation of roles and responsibilities across different phases of development (see Table 2). For example, an Under 9's coach working within the foundation phase, is likely to be presented with different daily challenges than an Under 21's coach due to the aims of the team, their context, the needs and wants of the players, and the key stakeholders involved within the players development. By extension, without a nuanced understanding of a coach's context, it will limit the ability for the Premier League to design coach development that presents a level of near transfer, that is designing tasks that closely represent the challenges of day-to-day coaching (40). Without this fidelity, genuine coach learning and its application to practice is likely to be limited.

**Table 2:** An example of age/stage specific considerations of skillful coaching taken from the Premier Leagues Elite Player Performance Plan (EPPP).

Phase of development	Foundation phase	Youth development phase	Professional development phase
Age Groups	Under-9 to Under-11	Under-12 to Under-16	Under-17 to Under-23
Coaching role specific contextual differences	Phase lead	Phase lead	Phase lead
	Age group head coach	Age group head coach	Age group head coach
		Age group assistant coach(es)	Age group assistant coach(es)
	Age group assistant coach(es)		
		Specialist coaches (e.g., goalkeeper coach).	Specialist coaches (e.g., individual development coach).

### Identifying what is missing—the strengths and limitations of coaching expertise research

One consequence of the lack of contextual focus has been the widespread use of competency frameworks that fail to prepare coaches for the realities of working with the complexity of human beings. A key problem of this approach being the focus on lists of “nice to haves”, focused on what coaches do, without reference to the typical challenges or problems that coaches might face in their context. For instance, a competency framework may include a statement such as; *demonstrate competence to ask clear and challenging questions to players to check for understanding*. These types of statements would serve to limit skilful coaching and expertise, as the coach is tasked with simply asking clear and challenging questions, without the deeper consideration of when, how and why a specific coaching style may be optimal to support player learning. Subsequently, there is an argument for coaching to move away from an over emphasis on routine competencies and towards the adaptive nature of problem-solving that skilful coaches need to engage in. This is not to suggest that competence is not a worthy aim, procedurally competent practitioners are a necessity, but this might be a focus for minimum standards. It is the ambition of the Premier League for the academy coaching workforce to be taken beyond mere competence and consider the need for expertise in practice.

Therefore, conceptually it would appear that the research is asking the right questions if we are to make this progression towards the recognition of expertise in practice (23, 24). However, this message has been reiterated for over two decades (52), with little indication of response by researchers. Empirical research which has investigated the nature of skilful coaching and expertise has failed to consider its contextual nature (53). Mirroring ongoing discussions in the leadership literature (54), this cross sport, individual focus ignores the need for coach capacities to be attuned to contextual demands. By conducting an empirical investigation into the practice of those deemed to possess expertise in academy football, there is an opportunity to initiate a long-desired paradigm shift, away from competencies and toward skill and expertise in coaching. To achieve this, we need to consider how we identify individuals with adaptive skill and expertise within Premier League Academies.

## How should we identify coaches with expertise across contexts?

Unfortunately, there is a historical trend in research across various fields of failing to justifiably identify individuals with expertise, including misrepresentation of the skilful due to a lack of appreciation of experience, skills and attributes, or peer validation (46). For practical and methodological reasons, much research in coaching has focussed on partial conceptions of a coach's role and responsibility with a sole focus on practice design, skill acquisition, or coaching behaviours on the pitch/court/track. Evidence suggests that a coach's role extends far beyond these situations, to classroom settings, conversations in corridors, pre or post sessions, all of which are largely ignored throughout the literature (55). Furthermore, as a field we have tended to over rely on cross-sectional, retrospective, and observational methods in isolation. This often constitutes a snapshot of coaching practice at a moment in time, without appreciation of context or socio-political considerations.

In contrast, other fields typically follow conceptions of expertise with empirical investigations to test and/or explore them. Thus, there is always a desire to build an evidence base which informs individuals to understand how these parameters might look, sound and feel, like in the challenges of day-to-day work. Unfortunately, this leaves us with limited evidence to inform skilful coaching. This is not, however, to say that the expertise literature from alternative domains cannot inform sport coaching. Recently Nash and colleagues (26) have stressed the importance of exploring research findings from other domains, and consider how data may, or may not, transfer into a different field. This presents an alternative approach away from the norms of research in sport coaching. Therefore, to further essential research questions in sport coaching, we now consider how this literature might act as a starting point.

### Identifying experts within PL academies

Across the domain specific expertise literature, a common thread of “capacities” have been identified as characterising expertise (56–58). Hoffman (46) summarised these capacities, suggesting transferability across domains and contexts (see our adaptation in Figure 2).

The capacities presented within Figure 2, are highly subjective, contextually applied and correspond with highly skilful practice. This contrasts with objective competencies as observable patterns of behaviour that need to be performed to fulfil a role competently (59). Based on the complexity of these capacities, a multidimensional and nuanced approach is needed to identify and recruit “skilful and adaptive” coaches for research. Attention should be placed on the significance of social capacities, where coaches' reputations across the workforce are a critical, but often ignored marker. This might be best captured by considering the disruption someone's absence from the coaching environment could cause, and the subsequent impact on its function (60, 61). Based on these broader recommendations, alongside the capacities presented in Figure 2, we suggest the below criteria to identify the most skilful coaches (see Table 3).

**Table 3:** Criteria designed to support the identification of the most skillful coaches within Premier League academies.

Self-reported capacities	Peer-reported capacities	System-reported capacities
Attempts to understand the knowledge base, skills, attributes and experiences of workforce.	Attempts to understand the capacities of skillful coaches from managers and peers within a system.	Attempts to understand the capacities of skilful coaches from individuals responsible for governing systems or competitions.
e.g., survey self-reported beliefs from coaches working in PL academies		
	e.g., asking key individuals to highlight who they believe to be the most skillful and why, such as the academy Head of Coaching.	e.g., central staff who have observed, heard or interacted with skillful coaches e.g., coach development workforce at the PL.

Furthermore, we strongly suggest that if authors choose to adopt the language of “expertise” or “most skilful” they should critically consider the courses of action and research that have led to it.

## What does coaching expertise research need to do?

Outside of sport coaching, expertise research has adopted a highly pragmatic focus, with the intent of informing practice (60, 61). The common feature of this pragmatic approach is identifying common and critical challenges of work first, then working backwards to the perceptual demands, skills and knowledge (*often packaged as coaching theories in the sport coaching field*) required to meet these challenges. Exemplifying quality in such approaches, Ward et al. (33) drew on Woods (62) work, to suggest a combination of four methodological approaches;

1. Research should be contextually situated using individuals who represent the population and context under consideration (63, 64) e.g., using an Under-10's academy coach to understand skilful coaching within the foundation phase.
2. Use cognitive task analysis methods (e.g., eliciting verbal reports of thinking, roles and responsibilities, action protocols, knowledge) that externalize performance and delivery, which allow researchers to understand internal processes (27, 43, 65, 66).
3. Use retrospective analyses and stimulated recall of critical incidents to reconstruct the dynamics and problem-solving strategies used to approach critical challenges using participant interviews and other data [e.g., critical decision method (67)].
4. Field observations, which identify coherence of intentions, what is espoused (what coaches say they do) and what is in use [coach actions; (11)].

Therefore, in the desire to understand what skilful coaching looks, sounds and feels like, and answer third and final question in this paper, these methods should be adopted, carefully developed, combined and employed over an appropriate length of time which allows a true appreciation of context (e.g., a competitive football season).

## Developing an agenda for skilful coaching expertise

Within this paper we have discussed a lack of empirical research designed to explore the capacities of expertise and how they are realised in practice. Drawing on other domains, it is clear that skilful practice and expertise, is underpinned by the capacity to adapt skillfully to the requirements of the task at hand. Therefore, by adapting Hoffman's (46) capacities of experts, we have developed an evidence informed criteria, designed to identify skilful coaches by privileging the views and beliefs of the wider coaching workforce across Premier League Academies. Finally, drawing together the aforementioned methodological insights, alongside previous conceptual work in sport coaching, and recommendations from broader expertise literature, we conclude this paper by proposing an evidence informed agenda aimed at uncovering the hallmarks of the most adept coaches in Premier League Academies (see Table 4). This plan represents a broader ongoing research plan for the Premier League to drive the progress of research informed practice to understand skilful coaching and adaptive expertise, in turn shaping a coach development agenda focused on developing adaptive and skilful coaches throughout academies (33). Ultimately, the aim being for the Premier League to develop the most skilful coaching workforce in the world.

**Table 4:** The premier leagues proposed research agenda to investigate adaptive expertise and skill in academy football coaching.

Purpose	Methodological considerations
Identify coaches with justifiably high levels of adaptive skill within and across contexts	*Apply the Hoffman criteria alongside the evidence informed criteria presented in* Table 3.
	*Triangulation with coaches from each of the three phases of development self-reports using the Brief expertise scale for sports coaching* (68).
	*Identify six coaches from the Foundation Phase, Youth Development Phase and Professional Development Phase working within Premier League Football Academies to take part in the following methods*.
Investigate the developmental influences of coaches	*Biography interviews with coaches to understand coaches’ developmental journeys and the knowledge, people, and experiences within them* (69)*.*
Investigate critical and common coaching challenges	*In situ observations* (70)*, Cognitive task analysis tools (CTA) and interviews.*
Further investigate the perceptual demands, knowledge required, and strategies used to tackle specific challenges	*In situ observation* (70)*, Critical decision method (CDM).*
Coach development	*Collation and embedding of findings in coach development*.

## Data Availability

The original contributions presented in the study are included in the article/Supplementary Material, further inquiries can be directed to the corresponding author.
